# The Effects of Endocrine Disruptors on Adipogenesis and Osteogenesis in Mesenchymal Stem Cells: A Review

**DOI:** 10.3389/fendo.2016.00171

**Published:** 2017-01-09

**Authors:** Marjorie E. Bateman, Amy L. Strong, John A. McLachlan, Matthew E. Burow, Bruce A. Bunnell

**Affiliations:** ^1^Center for Stem Cell Research and Regenerative Medicine, Tulane University School of Medicine, New Orleans, LA, USA; ^2^Department of Pharmacology, Tulane University School of Medicine, New Orleans, LA, USA; ^3^Department of Medicine, Tulane University School of Medicine, New Orleans, LA, USA

**Keywords:** endocrine disruptors, mesenchymal stem cells, adipogenesis, tissue engineering, tissue scaffolds, immunomodulation

## Abstract

Endocrine-disrupting chemicals (EDCs) are prevalent in the environment, and epidemiologic studies have suggested that human exposure is linked to chronic diseases, such as obesity and diabetes. *In vitro* experiments have further demonstrated that EDCs promote changes in mesenchymal stem cells (MSCs), leading to increases in adipogenic differentiation, decreases in osteogenic differentiation, activation of pro-inflammatory cytokines, increases in oxidative stress, and epigenetic changes. Studies have also shown alteration in trophic factor production, differentiation ability, and immunomodulatory capacity of MSCs, which have significant implications to the current studies exploring MSCs for tissue engineering and regenerative medicine applications and the treatment of inflammatory conditions. Thus, the consideration of the effects of EDCs on MSCs is vital when determining potential therapeutic uses of MSCs, as increased exposure to EDCs may cause MSCs to be less effective therapeutically. This review focuses on the adipogenic and osteogenic differentiation effects of EDCs as these are most relevant to the therapeutic uses of MSCs in tissue engineering, regenerative medicine, and inflammatory conditions. This review will highlight the effects of EDCs, including organophosphates, plasticizers, industrial surfactants, coolants, and lubricants, on MSC biology.

## Mesenchymal Stem Cells (MSCs)

**Figure 1:**
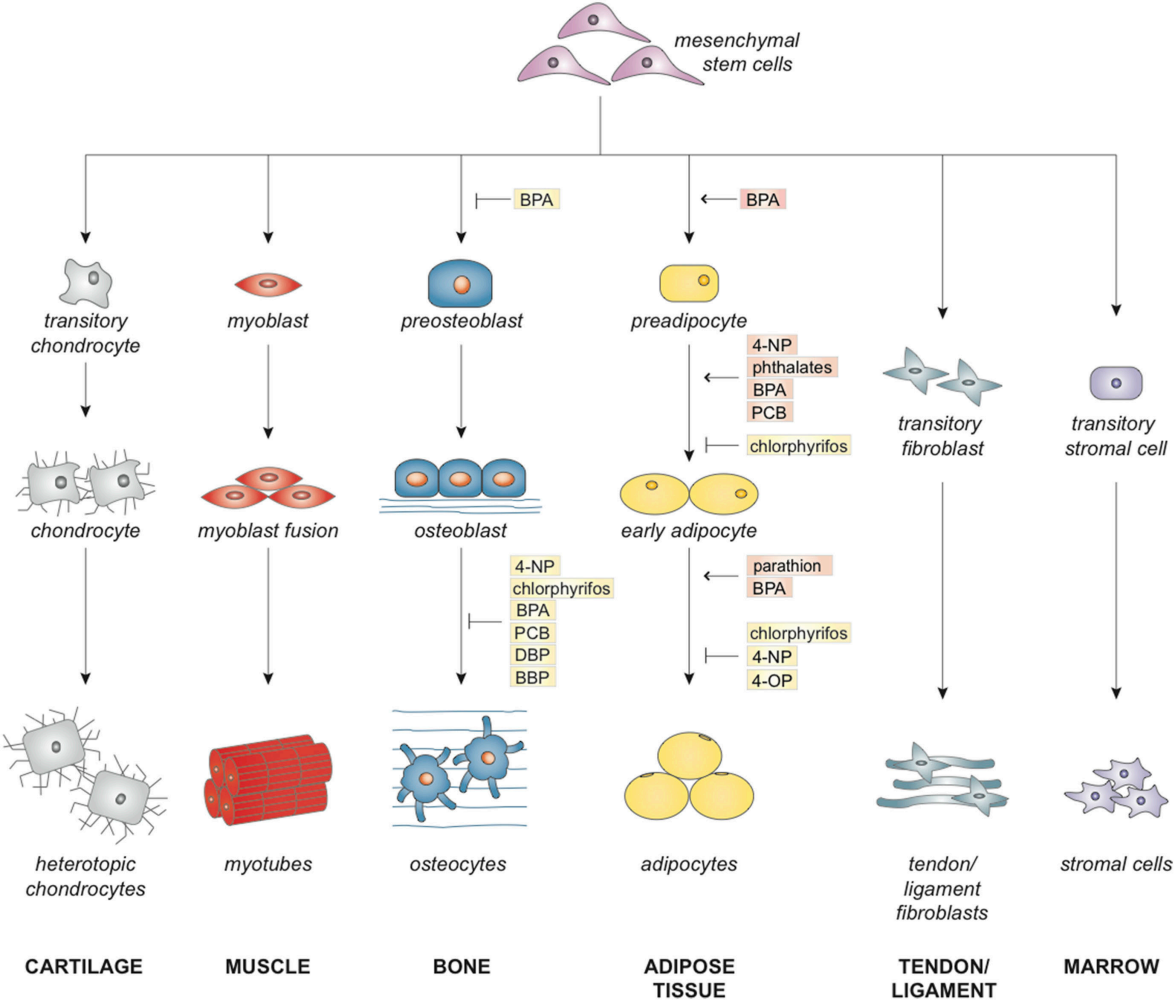
**Effects of endocrine disruptors on commitment and lineage-specific differentiation of mesenchymal stem cell**.

Mesenchymal stem cells are multipotent cells that maintain homeostasis in the human body by regeneration and repair of damaged and aged tissues. According to the International Society for Cellular Therapy, MSCs are cells that adhere to plastic in standard culture conditions, express surface antigens CD105, CD73, and CD90, lack hematopoietic antigens CD45, CD34, CD14 or CD11b, CD79alpha or CD19, and HLA-DR, and differentiate into chondroblasts, myoblasts, osteoblasts, adipocytes, fibroblasts, and stromal cells (1). Figure 1 depicts MSC differentiation into these lineages. An additional characteristic of MSCs is high self-renewal capacity, allowing these cells to retain their undifferentiated phenotype through senescence or until differentiation is induced (2). MSCs have been isolated from various tissues, including bone marrow, adipose tissue, periosteum, muscle tissue, blood vessels, lymphoid organs, skin, lung, umbilical cord blood, Wharton’s jelly, placenta, amniotic fluid, and fetal tissue (3–6).

With regard to tissue regeneration and repair, MSCs act by direct differentiation and paracrine signaling effects (4, 7–9). Under the appropriate stimuli, MSCs can differentiate into more specialized cells. Paracrine signaling effects of MSCs recruit other host cells and secrete growth factors and proteins to further stimulate regeneration to replace damaged cells (8). The wound-healing capacity of MSCs has led to studies in tissue engineering and regenerative medicine, such as seeding MSCs onto scaffolds to repair critical-sized bony defects (10). Scaffolds provide a three-dimensional structure to mechanically stimulate MSCs to undergo osteogenic differentiation or to secrete paracrine factors (10–13). Together, these studies suggest that MSCs may have potential applications in the repair of fractures and bony defects (10–13).

Mesenchymal stem cells have also been shown to reduce inflammation and have sparked significant interest due to their potential use in immunotherapy. Specifically, MSCs suppress T-cell proliferation and cytotoxic potential, inhibit maturation and T-cell stimulation by dendritic cells, inhibit B-cell proliferation and differentiation, reduce production of pro-inflammatory cytokines, such as tumor necrosis factor alpha (TNF-α), and enhance production of anti-inflammatory cytokines, such as interleukin 10 (IL-10) and interleukin 4 (8, 9, 14–18). These effects are mediated by release of immunomodulatory factors such as nitric oxide synthase (NOS), indoleamine 2,3-dioxygenase, prostaglandin E2, and IL-10 in MSCs (18). MSCs are capable of prolonging survival of allografts, reducing acute graft-versus-host disease, and improving outcomes in experimental autoimmune encephalomyelitis (15–19). Thus, MSCs may have broad therapeutic uses in the prevention and treatment of diseases with pro-inflammatory pathogenesis.

## Endocrine Disruptors

Endocrine-disrupting chemicals (EDCs) are environmental substances that alter the function of the endocrine system, producing adverse health effects in exposed organisms and their offspring (20). EDCs have been shown to have a variety of effects on MSCs. Recent studies gleaned from other cell types have also provided insight into the effects of EDCs on MSCs. These effects of EDCs may alter the therapeutic efficacy of MSCs and thus should be further elucidated.

## Effect of Endocrine Disruptors on MSCs and MSC Lineages

**Figure 2:**
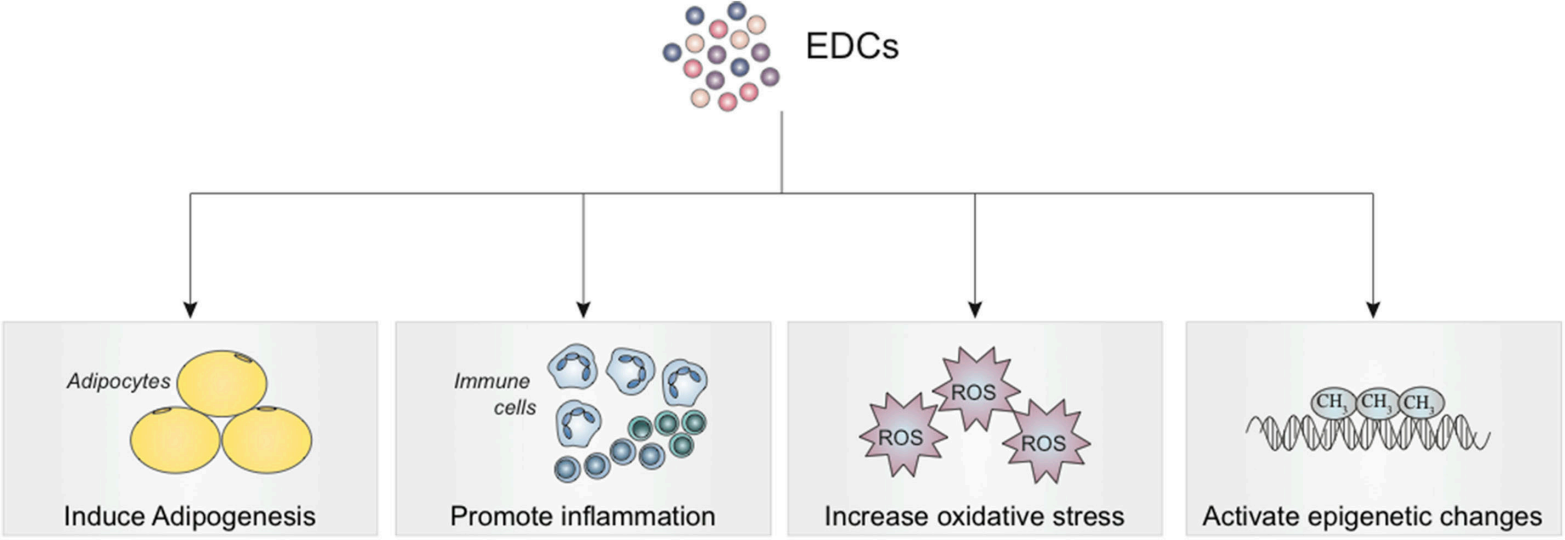
**Effects of endocrine-disrupting chemical exposure on mesenchymal stem cells with implications for tissue engineering, regenerative medicine, and treatment of inflammatory conditions**.

Low concentrations of several EDCs have been found in various human tissues. While these concentrations are as low as 100 pM to 1 nM, EDCs have been demonstrated to exert effects at these concentrations (21–23). Structural similarities between these EDCs and endogenous hormones indicate that the ability of EDCs to affect homeostasis may be through activation of hormone receptors. Like hormones, EDCs are able to function at very low doses in a tissue-specific manner, which is consistent with EDCs having non-monotonic dose–response curves (21–24). Therefore, the presence of low levels in human subjects does not indicate lack of harm from EDC exposure (21–23). At these levels, studies have shown that EDCs induce adipogenesis, increase oxidative stress, promote a pro-inflammatory state, and produce epigenetic changes (22). Low levels of EDCs have been shown to induce adipogenesis, increase oxidative stress, promote a pro-inflammatory state, and produce epigenetic changes. These effects are depicted in Figure 2.

## Adipogenesis

Adipogenesis is the differentiation of preadipocytes into adipocytes and is important for storage of lipids and metabolism in the human body. Adipogenesis requires a supportive environment and a peroxisome proliferator-activated receptor gamma (PPARγ) ligand (25). In order to support adipogenic differentiation, the appropriate cell density, spatial cell distribution, extracellular matrix, and a soluble hormonal stimulus, such as insulin-like growth factor 1 receptor, glucocorticoid receptor (GR), or cyclic adenosine monophosphate-dependent protein kinase must be present. As the master regulator of adipogenesis both *in vitro* and *in vivo*, PPARγ has been demonstrated to be necessary and solely sufficient for adipogenic differentiation to occur in a supportive environment (21, 26, 27). PPARγ expression, induced by CCAAT/enhancer-binding protein (C/EBP) β and δ, engages in a feed-forward loop with C/EBPα to promote adipogenesis (21, 26).

Endocrine-disrupting chemical exposure *in utero* and after birth has been linked to increased adipogenesis and the obesity epidemic. During development *in utero* and in the first few years of life, children are exposed to EDCs that can induce changes in stem cells during periods of differentiation and alter developmental programing of metabolism (21, 25, 28). These changes induced in MSCs, including epigenetic alterations, may predispose MSCs to undergo adipogenesis, leading to obesity later in life (25, 29–31). Continued lifelong exposure to EDCs may further exacerbate the situation by promoting adipogenesis and altering metabolism in a population already susceptible to obesity (32). *In vivo* studies of the effects of endocrine disruptors have confirmed the findings of *in vitro* studies. Studies in rats and mice have demonstrated increased body weight and visceral adiposity in animals exposed to EDCs (33–37). Perinatal and prenatal exposures have also been shown to result in excessive weight gain and adipose tissue mass in offspring (30, 31, 38–47). Human studies have demonstrated a positive association between EDC exposures and obesity, increased weight circumference, or increased body mass index (28, 30, 40, 42, 48–65).

The precise mechanism by which EDCs promote adipogenesis has been linked to PPARγ and promotion of a supportive environment for adipogenesis. Several EDCs have been shown to upregulate MSC and preadipocyte differentiation into adipocytes at concentrations ranging from 100 pM to 100 μM: dichlorodiphenyltrichloroethane or 1,1,1-trichloro-2,2-bis (*p*-chlorophenyl)-ethane (DDT), 4-nonylphenol (4-NP), octylphenol (OP), bisphenol A (BPA), polychlorinated biphenyl (PCB)-77, PCB-101, PCB-153, PCB-180, di-(2-ethyl hexyl)phthalate (DEHP), mono-(2-ethylhexyl)phthalate (MEHP), dibutyl phthalate (DBP), benzyl butyl phthalate (BBP), dicyclohexyl phthalate (DCHP), and mono-benzyl phthalate (MBzP) (30, 35, 37, 39, 40, 42, 43, 46, 63, 66–80). Many endocrine disruptors target PPARγ by binding to it directly to activate downstream cascades that lead to enhanced adipogenesis or by increasing PPARγ expression to allow for a lower threshold for activation. These EDCs include DDT, dichlorodiphenyldichloroethylene or 1,1-dichloro-2,2-bis (p-chlorophenyl)-ethylene (81), 4-NP, OP, BPA, PCB-77, DEHP, MEHP, DBP, BBP, and MBzP (35, 37, 39, 43, 63, 66, 68–70, 75, 77–80, 82–85). Perinatal exposure to 4-NP has also been shown to increase PPARγ gene expression and sterol regulatory element-binding factor 1 (SREBF-1) expression in adipose tissue (83). SREBF-1 is a key transcriptional activator involved in adipogenesis and transcription of *PPARG*, the gene encoding PPARγ. BPA has been shown to directly upregulate SREBF1. BPA has also been shown to upregulate mammalian target of rapamycin pathways in human preadipocytes, and the activation of this pathway through phosphoinositol-3 kinase/Akt leads to the activation of PPARγ and SREBF-1 (85, 86). Thus, PPARγ and SREBF-1 are key transcriptional factors in adipogenesis. The expression of C/EBPα and expression of factors promoted by PPARγ, such as lipoprotein lipase and fatty acid binding protein 4/adipocyte protein 2 (aP2), have been shown to be increased in response to DDT, DDE, 4-NP, BPA, PCB-77, DEHP, MEHP, and BBP (37, 39, 42, 45, 46, 66, 68, 69, 73, 75, 76, 78, 82–84, 86–89). *p*,*p*′-DDT has also been shown to increase binding of C/EBPα to its DNA response element, demonstrating that the promotion of adipogenesis may be occurring through both increased expression and activation of targeted receptors (66).

In order to promote a supportive environment for adipogenesis, studies have also shown that 100 pM to 1 µM of EDCs, such as BPA and DCHP, may directly or indirectly cause increased interaction with the GR. BPA and DCHP have been shown to act through the GR to increase lipid accumulation and adipogenesis (34, 67, 71). BPA also has GR-mediated indirect effects by increasing mRNA expression and enzymatic activity of 11beta-hydroxysteroid dehydrogenase 1. This enzyme converts cortisone to cortisol, which can bind the GR in adipose tissue and promote adipogenesis (46).

**Figure 3:**
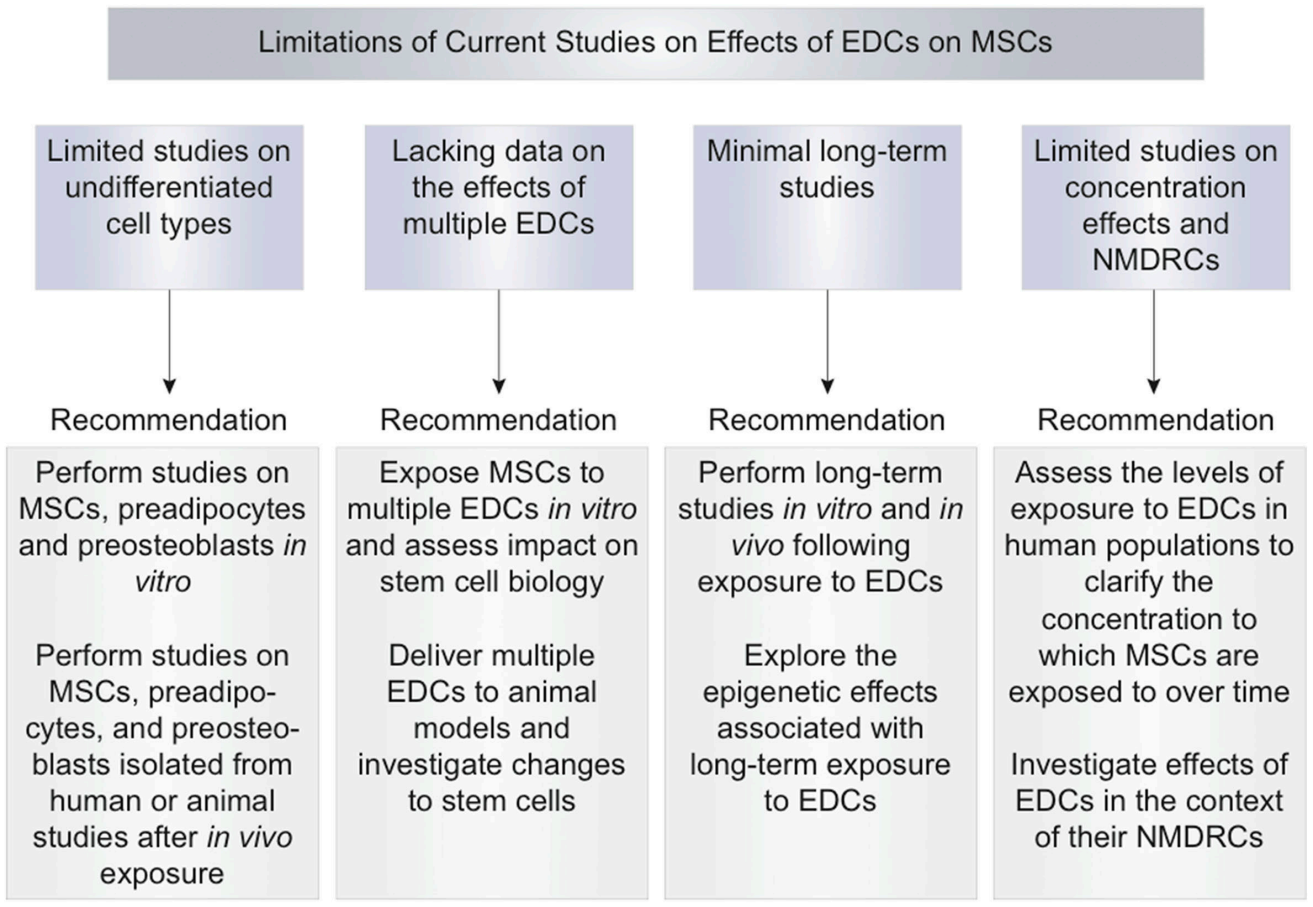
**Limitations of current studies of endocrine-disrupting chemical effects on mesenchymal stem cells and recommendations**.

In addition to PPARγ- and GR-mediated pathways, EDCs have been shown to enhance adipogenesis through other pathways. Paradoxically, 100 nM to 10 µM concentrations of DDT and BPA have the capacity to enhance adipogenesis by estrogen receptor (ER)-mediated signaling, which has classically been shown to inhibit adipogenesis (68, 69, 90–93). Biasiotto and colleagues addressed the issue of multiple endocrine disruptors simultaneously acting on MSCs in the environment (Figure 3). This study demonstrated that the combination of endocrine disruptors such as BPA and NP present at concentrations of 40 and 90 nM in wastewater may promote adipogenesis through ER-mediated pathways. Pure BPA at 50 and 80 µM also induced adipogenesis in this study (35). At 25 and 50 µM of BPA, induction of aP2, PPARγ, C/EBPα, and C/EBPβ expression has been shown to at least partially occur through a non-classical ER pathway (72). Interestingly, 4-NP exposure in mice has seemingly the opposite effects with the deletion and downregulation of ERα in adipose tissue, as increased adiposity due to fat cell differentiation was observed in mice (83).

Endocrine-disrupting chemicals can also increase adipogenesis in a paracrine manner by affecting soluble cues in the preadipocyte or MSC environment. Leptin, an anti-obesity hormone, has been shown to promote the use of metabolic fuels such as fatty acids rather than storage of fatty acids to form triglycerides. PCB-101, PCB-153, and PCB-180 at 1 µM concentrations have been shown to increase lipid accumulation and indirectly induce adipogenesis by inhibiting leptin (74).

Together, these results suggest that EDCs have the capacity to induce adipogenesis of MSCs and preadipocytes through increases in PPARγ signaling and alterations in the molecular environment. Increased adipogenic differentiation may lead to a reduced number of MSCs committing to the osteoblastic lineage and may reduce the ability of MSCs to undergo osteogenic differentiation (Figure 1). More studies should be performed with concentrations of EDCs in the picomolar to nanomolar range as EDCs may exert additional effects at these concentrations (21–23). Further limitations of the current studies in this field are the limited number of studies performed on undifferentiated, stem cells and the lack of data on the effects of exposure to multiple EDCs (Figure 3). Areas for improvement include implementation of more studies involving MSC exposure to EDCs and multiple EDCs simultaneously (Figure 3).

## Osteogenesis

Osteogenesis is the differentiation of MSCs into osteoblasts. Osteogenic differentiation is driven by Runx2, a transcription factor that regulates the expression levels of osteogenic genes. These genes include alkaline phosphatase (94), osteopontin, type I collagen, osteocalcin, and osterix (6, 94). There are several other signaling pathways involved in osteogenic differentiation, including bone morphogenetic protein (BMP), transforming growth factor beta (TGF-β), and Wnt/β-catenin signaling. Similar to adipogenesis, osteogenic differentiation relies on a mechanical stimulus with the appropriate growth surface stiffness, topography, tension, cytoskeletal organization, and soluble medium factors (95–99). A general principle is that stimulation of adipogenesis results in suppression of osteogenesis and vice-versa (76, 100–102). Thus, given the induction of adipogenesis by many EDCs, it is logical that EDCs have been shown to reduce the expression of genes and activity of transcription factors involved in MSCs undergoing osteogenic differentiation.

Several EDCs have been shown to reduce expression of Runx2 and other key osteogenic genes at varying stages of bone differentiation. Chlorpyrifos has been shown to inhibit osteogenesis in MSCs through inhibition of acetylcholinesterase, leading to an increase in acetylcholine (103). Increased acetylcholine has been shown to reduce ALP activity by nicotinic acetylcholine receptors and muscarinic acetylcholine receptors in preosteoblasts and osteoblasts, reducing osteogenic differentiation (104). MEHP has been shown to significantly suppress ALP activity, Runx2 expression, and osterix expression in MSCs (76). DEHP results in reduction of ALP expression, Runx2 protein levels, and mineralization in osteoblasts (105). The mechanisms by which 10 µM of MEHP and DEHP and 20 µM of MEHP inhibited osteogenesis were not determined in these studies, although Watt and Schlezinger and Bhat et al. demonstrated that the reduction in osteogenesis was not a result of decreased cell viability.

One proven mechanism of reduction of MSC osteogenesis by EDCs is their induction of apoptosis of osteoblast lineage cells. A total of 2.5 µM of p-NP has been shown to reduce osteogenesis of MSCs and to reduce cell viability (106). Aroclor 1254 and BPA have been reported to reduce osteogenesis of preosteoblasts and to reduce cell viability at concentrations ranging from 1 to 10 µM for Aroclor 1254 and from 2.5 to 12.5 µM for BPA (107, 108). A total of 1–10 µM concentrations of 4-NP, BBP, and DBP have also been shown to decrease viability of osteoblasts and preosteoblasts through promotion of apoptotic pathways (109, 110). Notably, the EDC effects in these studies occurred in a dose-dependent manner with higher doses of EDCs being more likely to result in reduced cell viability and decreased osteogenic differentiation. EDCs exert their effects over long-term environmental exposures.

One additional potential mechanism for the reduction in MSC osteogenic differentiation is the alteration of the cellular microenvironment through EDC reduction of serum estradiol levels. Estradiol has been shown to induce MSC differentiation toward an osteogenic lineage and to increase MSC expression of osteogenic genes including Runx2, ALP, collagen I, TGF-β1, and BMP2 (91, 111–114). EDCs including chlorpyrifos, OP, BPA, DEHP, MEHP, DBP, and mono-butyl phthalate (MBP) have been shown to reduce serum estradiol and testosterone levels (115–122). EDC reduction in the level of estradiol in the MSC microenvironment may cause MSCs to shift away from an osteogenic lineage and toward an adipogenic lineage. The reduction in serum estradiol and the mechanisms by which it may affect MSCs must be further investigated in future studies.

These studies suggest that EDCs may alter the capacity of MSCs to undergo osteogenic differentiation by reducing cell viability and altering cell microenvironment (Figure 1). A strength of the studies on the osteogenic effects of EDCs was the use of a treatment period that is classified as “long-term” or greater than or equal to 7 days. Long-term treatment periods more accurately mimic the effects of chronic environmental exposure to EDCs. Limitations of current studies of EDC inhibition of osteogenesis include lack of studies testing EDC concentrations in the picomolar to nanomolar range, lack of studies in MSCs and preosteoblasts, and lack of assessment of exposure to multiple EDCs simultaneously (Figure 3). Areas for improvement include implementation of more studies using low concentrations of EDCs, *in vitro* studies testing various EDCs in MSCs and preosteoblasts, *in vivo* studies of MSCs isolated from EDC-exposed animal subjects, and studies of exposure to multiple EDCs simultaneously (Figure 3).

## Oxidative Stress

Oxidative stress is an imbalance in the production of free radicals and detoxification by antioxidants. Oxidative stress can be measured by alterations in the levels and activity of antioxidant enzymes, such as superoxide dismutase, catalase, glutathione (GSH) peroxidase levels, the GSH/glutathione disulfide ratio, and malondialdehyde levels. These enzymes detoxify reactive oxygen species (ROS) by reducing them to water and other unharmful forms, preventing cellular damage and aging. Alterations in the levels of these enzymes can prevent the cell and organism from being adequately protected against ROS. Cellular aging is the decreased efficiency of function that occurs from damage from processes such as oxidative stress over time (123).

Oxidative stress has been associated with reduced self-renewal and early senescence of stem cells. While EDCs have been shown to induce ROS in a variety of cell types, studies have not been performed directly on MSCs. DEHP has been shown to promote oxidative stress and increase ROS in adipocytes (124). BPA has been shown to increase structural chromosome aberrations in bone marrow cells likely secondary to oxidative stress (125). ROS alter MSC biology by inhibiting osteogenesis, and increased ROS levels are associated with MSCs undergoing adipogenic differentiation (126). Additionally, MSCs exposed to ROS during expansion and MSCs from older subjects have reduced T cell suppression capacity due to alterations in MSC immunophenotype (126–128). Oxidative stress also affects the ability to expand MSCs in culture due to replicative senescence and reduced proliferation (126, 129). Therefore, it is essential to determine whether EDCs induce ROS in MSCs because ROS may induce changes in MSCs that may affect the ability to expand cells for use in therapy, alter differentiation ability, and reduce immunomodulatory capacity.

The effects of EDCs on ROS generation have not been well described. Limitations of current studies include lack of studies performed directly on MSCs, lack of studies testing various EDCs, lack of testing exposure to multiple EDCs simultaneously, and lack of long-term studies to assess the effects of chronic exposure to ROS. These limitations and areas of improvement to address in future studies are outlined in Figure 3.

## Pro-Inflammatory State

Inflammation is a localized response to tissue injury. EDCs including diazinon, parathion, malathion, BPA, PCB-77, PCB-153, and PCB-180 increase the levels of pro-inflammatory cytokines, such as TNF-α and interleukin 6 in adipose tissue (34, 37, 48, 71, 74, 81, 84, 88, 130–134). Together, these EDC effects induce a pro-inflammatory phenotype in adipose tissue. Animal studies have also demonstrated an increase in pro-inflammatory cytokines in adipose tissue following exposure to parathion and PCB-77 (48, 130).

The pro-inflammatory state induced by EDCs may fundamentally alter MSC biology. In general, MSCs suppress inflammation, and pro-inflammatory cytokines increase the immunomodulatory capacity of MSCs (135–137). While MSCs have immunosuppressive effects in the context of vigorous inflammation, recent studies have demonstrated that low-level inflammation or inhibited expression of immunosuppressive factors such as NOS can result in MSC induction of immune response (18, 138). The chronic, subacute exposure to EDCs, such as diazinon, parathion, malathion, BPA, PCB-77, PCB-153, and PCB-180, may induce low-level inflammation, leading to increased pro-inflammatory cytokines secreted by MSCs and induction of the immune response (18).

Pro-inflammatory effects of EDCs may also have an effect on MSC differentiation. Interestingly, pro-inflammatory conditions may increase expression of osteogenic genes such as ALP and result in increased mineralization (139, 140). Li et al. further demonstrated that conditioned medium from TNF-α-activated MSCs can enhance osteogenesis through paracrine mechanisms (139). However, Sidney and colleagues demonstrated decreases in cell viability and reduction in formation of bone nodules by primary osteoblasts in response to cytokine stimulation (141). These two opposing studies may be explained by differences in long-term and short-term exposures to pro-inflammatory conditions. In a study of long-term exposure to pro-inflammatory cytokines (TNF-α and interleukin 1 beta), stem cells from the apical papilla demonstrated inhibition of osteogenesis while in short-term culture, cytokines induced mineralization (142). A similar study in bone marrow MSCs demonstrated promotion of osteogenesis with short-term TNF-α exposure and inhibition of osteogenesis with long-term exposure (143). In these two studies, inhibition of osteogenesis occurred at greater than or equal to 7 days while promotion of osteogenesis occurred at time points less than 7 days. This is the reasoning behind our definitions of short-term and long-term exposure to inflammation throughout this review. The model of EDC effects is more likely to represent a long-term exposure and thus to decrease osteogenesis.

In summary, these studies indicate that long-term exposure to EDCs may result in MSC induction of a pro-inflammatory state that can inhibit osteogenesis. The primary strength of these studies stressed the long-term effects of EDCs and the differential effects of short and long-term exposure to EDCs. Limitations include lack of studies demonstrating induction of inflammatory state directly in MSCs and lack of studies on effects of exposure to multiple EDCs simultaneously. Future studies should investigate the pro-inflammatory state of MSCs after *in vitro* and *in vivo* EDC exposures (Figure 3). Additionally, the effects of multiple endocrine disruptors should be tested simultaneously in MSCs to improve understanding of environmental exposures of humans to multiple EDCs (Figure 3).

## Development of Epigenetic Changes

Epigenetic changes are alterations in gene activity that do not alter DNA sequence. EDCs can induce epigenetic changes in the undifferentiated cells of the fetus or in undifferentiated adult stem cells by oxidative stress or changes in ligand signaling. Epigenetic changes following EDC exposure include alterations in DNA methylation, histone acetylation, and microRNA (miRNA) expression. Several of the epigenetic changes induced by EDCs may explain their propensity to induce adipogenesis and inhibit osteogenesis. These epigenetic changes can also be passed to subsequent generations of stem cells and if present in the germline, can persist in subsequent generations of offspring (21, 23, 29, 31, 40, 42, 144).

Epigenetic effects of EDCs may be key in the induction of adipogenesis and the inhibition of osteogenesis in MSCs. The non-canonical Wnt/β-catenin pathway, which has been associated with osteogenic differentiation, has been shown to inhibit PPARγ transactivation by H3K9 methylation of its target genes (145). Inhibition of PPARγ results in a shift toward osteogenic differentiation while decreased methylation would activate PPARγ and shift toward adipogenic differentiation. BBP has been directly shown to cause histone modifications that induce MSCs to undergo adipogenic differentiation such as the enhancement of H3K9 acetylation, the increase of histone acetyltransferases such as p300 expression and GCN5 expression, the reduction of histone deacetylase expression, and the decreased dimethylation of H3K9 (75). Notably, these effects were seen at 100 nM to 50 µM concentrations of BBP with the decreased dimethylation effect occurring in the concentration range of 100 nM to 10 µM. Decreased trimethylation of histone H3K9(me3) and increased expression of miR-146a have also been shown in multiple cell types following exposure to BPA but have not yet been directly shown in MSCs (123, 146, 147).

Data regarding the epigenetic effects induced by EDCs are preliminary. *In vitro* studies on the epigenetic effects should specifically be performed in MSCs isolated from various tissues. Once *in vitro* EDC epigenetic effects in MSCs are clarified, MSCs should be isolated from human subjects of various ages and tested for EDC-specific epigenetic changes. These subjects should also have serum levels of EDCs tested at multiple time points to identify the concentration of EDCs to which human subjects and their stem cells are exposed over time. Studying the EDC-induced epigenetic changes in MSCs in humans exposed to EDCs present in the environment would provide information about cumulative, lifetime EDC exposures in potential MSC donors (Figure 3).

## Potential Implications of EDC Exposure on Therapeutic Potential of MSCs

### Effect on Tissue Engineering

The observed adipogenic effects and potential ROS-inducing effects of EDCs, such as DDT, BPA, alkylphenols, PCBs, and phthalates, on MSCs have important implications with regard to tissue engineering. The therapeutic efficacy of MSCs with regard to the repair of defects and fractures is twofold. MSCs must be able to differentiate into appropriate lineages, and they must be able to secrete appropriate paracrine factors that recruit other host cells and stimulate regeneration of the damaged tissue (4, 7–9).

One well-described effect of EDCs in the literature is the induction of adipogenesis and inhibition of osteogenesis in MSCs. In the context of tissue engineering for critical-sized defects and fractures, tissue scaffolds may be seeded with MSCs that have been exposed to EDCs, and the EDC exposure may impair the capacity of the MSCs for osteogenic differentiation. This leads to decreased bone formation, representing the impaired ability of MSCs to heal critical-sized defects and fractures. EDC-exposed MSCs may undergo adipogenesis, further reducing the ability of MSCs to directly regenerate damaged tissues and to promote wound healing. An additional point of consideration is that MSCs may be exposed to EDCs in the MSC donor or through high serum concentrations of EDCs in the MSC recipient. Thus, exposure of the MSC donor or the MSC recipient to EDCs may reduce the ability of the MSCs to promote wound healing by driving MSCs toward adipogenic differentiation.

In addition to induction of adipogenesis, EDC promotion of ROS production may reduce the capacity of MSCs to self-renew and differentiate, resulting in cellular senescence and aging. Studies of aged MSCs have demonstrated a reduced capacity for activation, migration, and differentiation (128, 148). Donors with greater lifetime exposure may have more aged MSCs, and seeding tissue scaffolds with these aged MSCs may result in a reduced capacity to regenerate damaged tissues and to recruit other cells to the site of injury. When determining an appropriate MSC donor source for promotion of wound healing, the lifetime exposure of the donor to EDCs should be carefully considered. A potential method for considering lifetime exposure is outlined in the Section “Development of Epigenetic Changes.”

Therefore, EDCs may reduce the therapeutic efficacy of MSCs in wound healing by inducing adipogenic differentiation and promoting ROS production. These EDC effects impair differentiation of MSCs into appropriate lineages for wound healing, MSC recruitment of other host cells, and stimulation of damaged tissue regeneration by MSCs. Future *in vivo* studies of tissue engineering should test the capacity of MSCs to regenerate damaged tissues and heal critical-sized defects in the context of EDC exposure. It is particularly essential to collect these data as animal subjects in tissue engineering studies may have natural levels of exposure to EDCs that do not accurately reflect the exposure of human subjects to EDCs.

### Effect on Immunomodulatory Capacity

The immunomodulatory capacity of MSCs is likely altered by exposures to EDCs, such as organophosphates, DDT, BPA, alkylphenols, PCBs, and phthalates. The ability to use MSCs to increase survival of allografts, reduce graft-versus-host disease, accelerate wound healing, and improve outcomes in demyelinating diseases is also twofold. MSCs suppress pro-inflammatory conditions and have various effects on the immune system (8, 9, 14–18). The therapeutic efficacy of MSCs is also contingent upon the lack of immunogenicity of the MSCs themselves to prevent pro-inflammatory reaction upon treatment.

The effects of EDCs in altering immunomodulatory capacity of MSCs include the promotion of oxidative stress and induction of adipogenesis. MSCs exposed to oxidative stress and MSCs which have undergone cellular senescence have shown reduced ability to suppress inflammation (148). Therefore, donor MSCs that have been aged by exposure to EDCs may have less therapeutic effect in conditions such as multiple sclerosis. Additionally, EDC-induced ROS production may upregulate adipogenic differentiation in MSCs. MSCs undergoing adipogenesis have been demonstrated to have a slightly different immunophenotype and secretome from MSCs not committed to a lineage, which could result in increased immunogenicity upon treatment (149, 150).

The chronic, subacute inflammatory state induced by EDCs has further been shown to increase secretion of pro-inflammatory cytokines by MSCs (8, 18, 151, 152). This could lead to impaired wound healing, acute graft loss, worsening of graft-versus-host disease, and worsened outcomes in demyelinating diseases. However, MSCs have also been shown to have stronger immunosuppressive effects in the context of higher levels of inflammation, levels which may be present in autoimmune diseases. It is possible that upon inoculation, donor MSCs may be exposed to a sufficient level of inflammation to induce their immunosuppressive properties.

Therefore, EDCs impair the capacity of MSCs to immunomodulate pro-inflammatory conditions by inducing adipogenesis, promoting oxidative stress, and causing a chronic, subacute pro-inflammatory state. These changes may result in reduced immunosuppression and increased immunogenicity of MSCs. Further studies are needed *in vivo* that examine the immunomodulatory capacity of MSCs following EDC exposure. It is possible that animals are not exposed to the same levels of EDCs and thus are not accounting for potentially reduced capacity of MSCs to improve outcomes in pro-inflammatory conditions following the exposure to EDCs.

## Conclusion

Endocrine-disrupting chemicals may alter the therapeutic potential of MSCs by effects on MSC differentiation capacity and biologic properties, including induction of adipogenesis, inhibition of osteogenesis, increase in oxidative stress, and promotion of a pro-inflammatory state. These effects may lead to reduced capacity of MSCs to differentiate into appropriate lineages and to induce paracrine signaling in wound healing. Additionally, they may decrease immunomodulatory effects by MSCs. The implications for tissue engineering and treatment of pro-inflammatory conditions are concerning and should be further explored with *in vivo* exposures to EDCs in animal subjects and studies of these potential effects on therapeutic efficacy. All of the alterations in MSC biology that result in changed therapeutic potential may ultimately be rooted in epigenetic alterations induced by EDCs that remain to be clarified, so future *in vitro* and *in vivo* studies should also explore epigenetic effects of EDCs on MSCs isolated from various tissues in the body. EDC-induced effects on MSCs should be considered when analyzing results of previous studies and should be further explored in future studies to more fully understand the implications for MSC therapies.

## Author Contributions

ME Bateman participated in the literature searches, design, writing, and editing of this review. AS participated in design, writing, and editing of the review. JM and ME Burow participated in the design and editing of this review. BB participated in the design, writing, and editing of this review.

## Conflict of Interest Statement

The authors declare that the research was conducted in the absence of any commercial or financial relationships that could be construed as a potential conflict of interest.

## Abbreviations

ALP, alkaline phosphatase; aP2, adipocyte protein 2; BBP, benzyl butyl phthalate; BMP, bone morphogenetic protein; BPA, bisphenol A; C/EBP, CCAAT/enhancer-binding protein; DBP, dibutyl phthalate; DCHP, dicyclohexyl phthalate; DDE, dichlorodiphenyldichloroethylene or 1,1-dichloro-2,2-bis (p-chlorophenyl)-ethylene; DDT, dichlorodiphenyltrichloroethane or 1,1,1-trichloro-2,2-bis (p-chlorophenyl)-ethane; DEHP, di-(2-ethyl hexyl)phthalate; DEP, diethyl phthalate; EAE, experimental autoimmune encephalomyelitis; EDC, endocrine-disrupting chemical; ER, estrogen receptor; GR, glucocorticoid receptor; IL-10, interleukin 10; MBP, mono-butyl phthalate; MBzP, mono-benzyl phthalate; MEHP, mono-(2-ethylhexyl)phthalate; miRNA, microRNA; MSC, mesenchymal stem cell; NMDRC, non-monotonic dose-response curve; NOS, nitric oxide synthase; NP, nonylphenol; OP, octylphenol; PPARγ, peroxisome proliferator-activated receptor gamma; PCB, polychlorinated biphenyl; ROS, reactive oxygen species; SREBF, sterol regulatory element binding factor; TGF-β, transforming growth factor beta; TNF-α, tumor necrosis factor alpha.
